# Pharmacological Effects of Novel Peptide Drugs on Allergic Rhinitis at the Small Ribonucleic Acids Level

**DOI:** 10.3389/fgene.2020.560812

**Published:** 2020-09-11

**Authors:** Li-Feng An, Zhan-Dong Li, Lin Li, Hao Li, Jian Yu

**Affiliations:** ^1^Department of Otorhinolaryngology Head and Neck Surgery, China-Japan Union Hospital of Jilin University, Changchun, China; ^2^College of Food Engineering, Jilin Engineering Normal University, Changchun, China; ^3^Measurement Biotechnique Research Center, Jilin Engineering Normal University, Changchun, China

**Keywords:** allergic rhinitis, peptide drugs, small RNAs, high-throughput sequencing, gene ontology, KEGG pathway

## Abstract

Using an allergic rhinitis (AR) model, we evaluated the pharmacological effects of novel peptide drugs (P-ONE and P-TWO) at the small RNA (sRNA) level. Using high-throughput sequencing, we assessed the sRNA expression profile of the negative control, AR antagonist (positive control), P-ONE, and P-TWO groups. By functional clustering and Gene Ontology and KEGG pathway analyses, we found that sRNA target genes have a specific enrichment pattern and may contribute to the effects of the novel peptides. Small RNA sequencing confirmed the biological foundations of novel and traditional AR treatments and suggested unique pharmacological effects. Our findings will facilitate evaluation of the pathogenesis of AR and of the pharmacological mechanisms of novel peptide drugs.

## Introduction

Allergic rhinitis (AR) (Maoz-Segal et al., 2019) is defined as inflammation of the nasal mucosa induced by an allergic reaction; it is also known as anaphylaxis (allergy) (Turner et al., 2019). Based on the clinical symptoms, AR can be classified into four groups based on its persistence and severity (Settipane and Charnock, 2016; Jung et al., 2020). For instance, AR of > 4-week duration is classified as persistent and AR with only mild symptoms as mild. These classifications can be combined; for instance, mild persistent AR (Settipane and Charnock, 2016). According to an independent survey, one in five people in Australia (Smith et al., 2017; Price et al., 2018) and one in three in the United States (Han et al., 2016) suffer or have suffered from AR, typically accompanied by asthma and allergic complications (Hill et al., 2016). Similar frequencies have been reported in other countries (Cardell et al., 2016; Bousquet et al., 2018). Therefore, AR is an important threat to human health globally.

Initially, the pathogenesis of AR was evaluated based on its pathological characteristics, such as inflammation and bacterial infection (Ledford and Lockey, 2016). However, these symptoms are similar to those of other diseases such as infectious rhinitis (the common cold) (Meltzer et al., 2000), indicating that phenotypic features cannot explain the differential susceptibility among populations. Next-generation sequencing enables genomic and transcriptomic analysis of disease. Polymorphisms of genes such as FcγRIIIa (Zeyrek et al., 2008) and those encoding histamine-metabolizing enzymes (García-Martín et al., 2007) are reported to be functionally related to the onset of such diseases. Moreover, the expression of some microRNAs (miRNAs; e.g., miR-370, miR-539, and miR-299) is altered during AR pathogenesis (Specjalski et al., 2016).

Histamine H4 receptor, a member of the G protein-coupled receptor superfamily, is a core regulatory factor of AR (Takahashi et al., 2009; Broide, 2010; Shiraishi et al., 2013). During the pathogenesis of AR, H4 receptor is upregulated, triggering immune over-activation (Lundberg et al., 2011; Walter et al., 2011) and remodeling of the inflammatory microenvironment by modulation of IL-6 and INF-γ expression (Peng et al., 2019). Among the drugs targeting the core pathogenic processes of AR, many target the H4 receptor. Indeed, two vaccines developed based on the immunological epitopes of the H4 receptor were effective in animal models (Wang et al., 2018). Such vaccines trigger an immune response against abnormally expressed H4 receptor. Th2 cells and IgE have been demonstrated to contribute to AR pathogenesis and the efficacy of H4 receptor-based therapeutics (Wang et al., 2018; Peng et al., 2019); however, the underlying biological mechanisms are unclear.

At the methodological level, in this study, we focused on two major biological/bioinformatics techniques: (1) establishment of guinea pig allergic rhinitis model; (2) analyses on small RNA sequencing data. For the establishment of guinea pig allergic rhinitis, researchers from multiple countries have developed and modified various methods to establish stable, reproducible and comparable models to mimic the pathogenesis of allergic rhinitis in human beings. In 2006, researchers from University of British Columbia (Canada) summarized the general workflow for the establishment of stable guinea pig allergic rhinitis model using ovalbumin (Al Suleimani et al., 2007), which is one of the most common allergens for guinea pigs’ respiratory tracts. In the next decades, the detailed techniques have been gradually modified but the major establishment procedure remains stable, implying the stability and efficiency of such ovalbumin based methods. Apart from that, another major methodological challenges for our study turns out to be the comparable small RNA sequencing analyses. With the development of computational methods, a general workflow for small RNA sequencing analyses has already been established including small RNA clustering, novel small RNA discovery, miRNA target prediction, differential expression of small RNA, evolutionary analysis, and functional analysis (Baran-Gale et al., 2015; Fuchs et al., 2015; Buschmann et al., 2016). In this study, we applied the latest workflow/software for small RNA identification and annotation [miRDeep2 (Friedländer et al., 2008) and RIPmiR (Breakfield et al., 2012)], revealing a robust small RNA profiling results for further analyses and summarization.

MicroRNAs are important in the pathogenesis of AR. In this study, miRNA profiling and a guinea pig model of AR enabled identification of the therapeutic mechanisms of two epitopes of the H4 receptor. All in all, based on the microRNA profiling techniques and blood samples from guinea pig model of allergic rhinitis (AR), we focused the underlying therapeutic mechanisms of two reported epitopes against H4 receptor for allergic rhinitis treatment at the microRNA level, trying to reveal their potential pharmacological mechanisms by targeting H4 receptors.

## Materials and Methods

### Reagents and Instruments

The following reagents were used: TRIzol (Invitrogen, 15596018), DEPC water (Ambion, AM9915), chloroform, isopropanol, and isoamyl alcohol (Xilong Chemistry). The following instruments were used: cryogenic centrifuge (Eppendorf), vortex oscillator (Qilinbeier), and TissueLyser II (Qiagen).

### Animal Models

We used 38-week-old male guinea pigs (Changchun Biological Products Research Institute Co., Ltd.; SCXK (Ji) 2016-0008) to establish a model of AR.

### Model Establishment

Following widely reported rhinitis guinea pig model establishment protocol (Narita et al., 1998), we established a guinea pig model of AR using ovalbumin (OVA). OVA causes less irritation and fewer side effects than toluene diisocyanate but is prone to degeneration or coagulation and so must be made fresh immediately before use.

### Small Peptide Screening

We first purified anti-HRH4 monoclonal IgG to a high purity (95%) for phage peptide library screening. Using HR4 antibody as the antigen, we screened out two peptides with high affinity for the monoclonal HRH4 IgG; these were named P-ONE (FNKWMDCLSVTH) and P-TWO (TFKFTLSYRQVH) and have been patented (Patent 1: “Vaccine based on mimicking human histamine receptor 4 (HR4) epitope and construction method thereof”, Application No.: 201510382851.1, Publication No.: 105017385B and Patent 2: “Using a phage antibody library to screen human histamine receptor 4 (HR4) epitope mimetic peptides and a vaccine construction method”, Application No.: 201510382781.X, Publication No.: 105037499B).

### Preparation of Vaccines

The peptide and CTB were dissolved in physiological saline, and the same volume of liposome Lipofect was added such that each 200 μL contained 100 μg of peptide and 5 μg of CTB. The mixture was stored overnight at 4°C and on the following day was brought to room temperature.

(1) Add 1 mL of saline to the antagonist to make a 25 mg/mL solution.
(2) Add 50 μL of normal saline to CTB to make a 10 mg/mL solution.
(3) The antagonist requires a total of 200 μL of nasal drops and is formulated as follows: Antagonist (peptide, 100 μg) 4 μL, CTB (5 μg) 0.5 μL, normal saline 95.5 μL, and liposomes 100 μL.
(4) P-ONE 20.7 mg, P-TWO 20.5 mg, and control vaccine 20.5 mg. Normal saline (NS) is added to P-ONE, P-TWO, and control vaccine as shown in Table 1.

**TABLE 1:** Vaccine preparation for different experimental and control groups.

Groups	Peptide (uL)	CTB (uL)	Physiological saline (uL)	Liposome (uL)
Negative Control	NA	NA	75	75
Positive Control	3.659 (JNJ77777120)	0.375	71	75
P-ONE	3.623 (P-ONE)	0.375	71	75
P-TWO	3.659 (P-TWO)	0.375	71	75

### Evaluation of Animal Model of AR

There is no uniform standard for the evaluation of AR models. Instead, such models are evaluated based on their ability to repeatedly trigger an allergic reaction.

Symptoms of AR, combined with changes in animal behavior and characteristic pathomorphological changes, were assessed to evaluate the model. After stimulation, animals with AR exhibit symptoms such as sneezing, scratching the nose, scratching the face, and a running nose. Most prior studies adopted the symptom score of Zhao (1993). We tested the model by evaluating sniffing, sneezing, and nasal discharge. During the evaluation, the superimposed quantitative score was applied to indicate the success of modeling. Symptom score is tested and calculated at the last time. After stimulation, each animal was observed for 30 min. The scoring criteria were as follows:

(1) Nasal itching: 1 point for one or two instances of light nose blowing, 2 points for moderate scratching of the nose/face, and 3 points for violent scratching of the nose/face.
(2) Sneeze: 1 point for 1–3, 2 points for 4–10, and 3 points for ≥ 11.
(3) Clearing the nose: 1 point for nostril flow, 2 points for the front nostril, and 3 points for the runny surface.

The three symptom scores were summed and a total score of ≥ 5 was considered a success. This experiment is based on observation records and combined with related behavioral indicators, verifying the success of the model.

### RNA Sampling

After the last dose on day 85, behavioral indicators were evaluated. We extracted RNA from blood samples for miRNA sequencing using the RNeasy Plus Micro and Mini Kits (Qiagen).

We fragmented and digested tissue samples by two methods. The first method is a lapping machine-based method. An appropriate amount of tissue sample was placed in a numbered grinding and crushing tube and 1.5 mL of TRIzol lysate was added. The mixture was ground in a TissueLyser II grinder for 30 s, and allowed to stand for 5 min. The second method was performed using liquid nitrogen. TRIzol lysate (1.5 mL) was transferred into a 2 mL EP tube. An appropriate amount of tissue sample was ground into powder in liquid nitrogen, transferred to the lysate, and allowed to stand flat for 5 min. Next, the disrupted tissue samples were centrifuged at 4°C and 12,000 g for 5 min. The supernatant was transferred to an EP tube containing 300 μL of chloroform: isoamyl alcohol (24: 1), mixed by inverting and shaking vigorously, and centrifuged at 12,000 *g* at 4°C for 8 min. If the middle layer was thick and the water phase turbid, extraction was repeated using the same volume of chloroform: isoamyl alcohol (24: 1).

The supernatant was transferred to a centrifuge tube containing 600 μL of isopropanol. Do not suck into the middle layer (micro-tissue or micro-cell sample, add 2 μL of 5 mg/mL glycogen-assisted precipitation during precipitation), mixed by inversion, and placed at −20°C for ≥ 2 h. Next, the sample was centrifuged at 17,500 *g* for 25 min at 4°C, the supernatant was discarded, and the pellet was washed with 0.9 mL of 75% ethanol and invert the suspended pellet. The sample was centrifuged at 4°C for 3 min at 17,500 *g* (depending on the precipitation), washed with 75% ethanol, and centrifuged at 17,500 *g* for 3 min at 4°C. The supernatant was discarded, residual liquid was removed after brief centrifugation, and allowed to dry for 3–5 min. Finally, the pellet was dissolved in 30–200 μL DEPC or RNase-free water.

### Small RNA Library Construction

We used the Agilent 2100 Bioanalyzer to evaluate sample integrity and concentration, and NanoDrop to detect inorganic ions or polycarbonate contamination.

To construct an RNA library, 0.2–1 μg of RNA was subjected to electrophoresis, 18–30 nt bands were selected (14–30 ssRNA Ladder Marker, TaKaRa) stripe and recycle. Next, we prepared a connection 3′ adaptor system at 70°C for 2 min and 25°C for 2 h and added RT primer at 65°C for 15 min followed by a ramp to 4°C at 0.3°C/s. Finally, we added the 5′ adaptor mix system at 70°C for 2 min and 25°C for 1 h. For reverse transcriptase-polymerase chain reaction (RT-PCR), we used First-Strand Master Mix and Super Script II (Invitrogen) and performed reverse transcription at 42°C for 1 h and 70°C for 15 min. Next, several rounds of PCR amplification using a PCR Primer Cocktail and Master Mix were performed at 95°C for 3 min; followed by 15–18 cycles of 98°C for 20 s, 56°C for 15 s, and 72°C for 15 s; followed by 72°C for 10 min; and a hold at 4°C. The PCR products were purified by electrophoresis and dissolved in EB.

The double-stranded PCR products were heat denatured and circularized by the splint oligo sequence. The single-stranded circular DNA (ssCir DNA) was used as the final library. The library was validating using an Agilent Technologies 2100 Bioanalyzer. The library was amplified with phi29 to generate a DNA nanoball (DNB), which harbored > 300 copies of one molecule. The DNBs were loaded into the patterned nanoarray and single-end 50-base reads were generated by combinatorial probe-anchor synthesis (cPAS).

### Small RNA Sequencing and Analysis

#### Data Filtering

The impurities in raw data include 5′ primer contaminants, no-insert tags, oversized insertion tags, low-quality tags, poly-A tags, small tags, and tags lacking a 3′ primer. Generally, an adaptor contaminant is caused by low sample quality or adaptor or sample concentration. The higher the adapter proportion, the greater the contamination. Low-quality tags are those with > 4 bases and a quality of < 10 or those with > 6 bases and a quality of < 13.

The above contaminant tags were removed, and the length distribution of clean tags was analyzed to evaluate sample composition. Small RNAs (sRNAs) are typically 18–30 nt in length (miRNAs, 21 or 22 nt; small interfering RNAs [siRNAs], 24 nt; and PIWI-interacting RNAs [piRNAs], 30 nt). The data were processed by removing tags of low quality, with 5′ primer contaminants, lacking a 3′ primer, without insertions, with poly-A, and of < 18 nt. The length distribution of the clean tags was summarized. After filtering, the remaining clean tags were stored in FASTQ format (Cock et al., 2009).

#### Reads Mapping

In general, the higher the alignment ratio, the closer the genetic relationship between the sample and the reference species. A low rate may be due to low similarity with the reference genome or to contaminants. Bowtie (Langmead et al., 2009) was used to map clean reads to the reference genome and to other sRNA databases. Please note that for Rfam we used cmsearch (Nawrocki and Eddy, 2013) with the default parameters.

#### Small RNA Classification

When annotating, some sRNA tags may be mapped to more than one category. To ensure that each sRNA was mapped to only one category, we used the priority miRNA > piRNA > small nucleolar RNA [snoRNA] > Rfam > other small RNA.

#### Small RNA Prediction

We used miRDeep2 (Friedländer et al., 2008) (for animals) and RIPmiR (Breakfield et al., 2012) (for plants) to predict novel miRNAs by exploring the characteristic hairpin structure of miRNA precursors. Piano (Wang et al., 2014), which is based on the support vector machine (SVM) (Scholkopf and Smola, 2001) algorithm and transposon interaction information, was used to predict piRNAs. The SVM classifier can be used in a wide range of species including human, mouse, rat, fruit fly, and insects. siRNA is a 22–24 nt double-strand RNA, one strand of which is 2 nt longer than the other. Due to this structural feature, we aligned tags to identify sRNAs meeting that criterion. Such tags were regarded as siRNA candidates.

#### Small RNA Expression

The sRNA expression level was calculated by the transcripts per million kilobases (TPM) method (John et al., 2004), which eliminates the influence of sequencing discrepancy. The data can be used for comparing gene expression between samples. To calculate the TPM the following formula was used:

(1) $$\mathrm{TPM}=\frac{C*10^{6}}{N}$$

#### Target Prediction

To identify targets we used RNAhybrid (Krüger and Rehmsmeier, 2006), miRanda (John et al., 2004), or TargetScan (Maziere and Enright, 2007; Agarwal et al., 2015) for animal, and psRobot (Wu et al., 2012) or TargetFinder (Fahlgren and Carrington, 2010) for plants. The default parameters were as shown in Table 2.

**TABLE 2:** Default parameter for target prediction.

Methods	Parameter
miRanda	-en -20 -strict
RNAhybrid	-b 100 -c -f 2,8 -m 100000 -v 3 -u 3 -e -20 -p 1 -s 3utr_human
psRobot	-gl 17 -p 8 -gn 1
TargetFinder	-c 4

#### Screening of DESs (Differential Expressed Sequences)

RNA sequencing could be modeled as a random sampling process, in which each read is sampled independently and uniformly from every possible nucleotide in the sample (Jiang and Wong, 2009). Under this assumption the number of reads from a gene (or transcript isoform) follows a binomial distribution (and can be approximated by the Poisson distribution).

Using the statistical model described above, DEGseq (Wang et al., 2009) proposes a novel method based on the MA-plot, a statistical analysis tool used to detect and visualize intensity-dependent ratios of microarray data (Yang et al., 2002). Let C1 and C2 denote the counts of reads mapped to a specific gene obtained from two samples, with Ci∼binomial (ni, pi), *i* = 1,2, where ni denotes the total number of mapped reads and pi the probability of a read coming from that gene. We define *M* = log2C1 - log2C2, and *A* = (log2C1 + log2C2 2). It can be shown that under the random sampling assumption the conditional distribution of M given that *A* = a (a is an observation of A) follows an approximately normal distribution. For each gene on the MA plot, we perform the hypothesis test H0: p1 = p2 versus H1: p1 ≠ p2. A *P*-value is assigned based on the conditional normal distribution.

The *P*-values calculated for each gene are adjusted to *Q*-values for multiple testing corrections by two strategies (Benjamini and Hochberg, 1995; Storey and Tibshirani, 2003). To improve accuracy, we defined a differentially expressed gene (DEG) as a fold-change of ≥ 2 and *Q*-value of ≤ 0.001. RNA-seq experiments have low technical background noise and the Poisson model fits the data well. In such cases, the technical replicates can be pooled to increase the sequencing depth and detect subtle changes in gene expression. Otherwise, a method that estimates noise by comparing the replicates is recommended.

#### Screening of DESs (Poisson Distribution)

Based on a prior report (Audic and Claverie, 1997), BGI (Beijing Genomics Institute) developed an algorithm to identify DEGs between two samples. If x is defined as the number of reads from sRNA A, x yields the Poisson distribution:

(2) $$p\left(x\right)=\frac{e^{\lambda }\lambda^{x}}{x!}\left(\lambda \mathrm{is}\mathrm{the}\mathrm{real}\mathrm{transcripts}\mathrm{of}\mathrm{the}\mathrm{gene}\right)$$

(3) $$2\sum_{i=0}^{i=y}p\left(i|x\right)$$

Or

(4) $$2*\left(1-\sum_{i=0}^{i=y}p\left(i|x\right)\right)\left(if\sum_{i=0}^{i=y}p\left(i|x\right)>0.5\right)$$

(5) $$p\left(y|x\right)=\left(\frac{N_{2}}{N_{1}}\right)y\frac{\left(x+y\right)!}{x!y!{\left(1+\frac{N_{2}}{N_{1}}\right)}^{\left(x\left|y\right|1\right)}}$$

In the equation above, the *P*-value of the differential gene expression test is corrected by the Bonferroni method (Atkinson, 2002). DES analysis is then performed on the sample; however, this generates thousands of hypotheses simultaneously (only if gene x is differentially expressed between the two groups); therefore, correction for false positive (type I errors) and false negative (type II) errors is performed by the false discovery rate (FDR) method (Benjamini and Yekutieli, 2001). In the next step, it is assumed that we have selected R DEGs among which S genes show differential expression, and the V genes are false positives. The error ratio (Q) is as follows: *Q* = V R. The user sets a cutoff value for Q (e.g., BGI sets a default cutoff of 5%), and the FDR is preset to < 0.05. To assess the significance of differences in gene expression, an FDR of ≤ 0.001 and an absolute Log2Ratio value of ≥ 1 are set as the default thresholds. More stringent criteria, such as a smaller FDR and larger fold-change value, can also be used to identify DEGs.

Next, we performed multiple hypothesis tests for the *P*-value of the differential gene expression test and determine the *P*-value field by controlling the FDR result. The conditions were set in advance so that the FDR cannot exceed 0.05. We also calculated the gene expression level (FPKM value) to assess differences in gene expression between samples. The smaller the FDR value, the greater the difference multiple, indicating a significant difference in expression. Genes with an FDR ≤ 0.001 and multiples of more than two-fold were regarded as differentially expressed.

#### Hierarchical Clustering Analysis

We performed hierarchical clustering of differentially expressed miRNAs using R package “pheatmap” (Kolde and Kolde, 2015). For more than two groups, hierarchical clustering of the intersection was performed, followed by union DESs.

#### Gene Ontology Enrichment Analysis

Gene Ontology (GO) (The Gene Ontology Consortium, 2016) is an international standard gene functional classification system. It offers a dynamically updated and controlled vocabulary, as well as a defined concept to comprehensively describe properties of genes and their products. GO has three ontologies: molecular function, cellular component, and biological process. The basic unit of GO is the GO term; each term belongs to a type of ontology.

GO enrichment analysis finds all GO terms that are significantly enriched in a list of DES target genes and finds genes that correspond to specific biological functions. To perform this analysis, BGI first maps all genes to GO terms in the database^1^, which calculates the number of genes for each term. The hypergeometric test is then performed to identify significantly enriched GO terms in the input gene list. The analysis was based on GO::TermFinder^2^ and was performed using the following algorithm:

(6) $$P=1-\sum_{i=0}^{m-1}\frac{\left(\begin{array}{c} M\\ i \end{array}\right)\left(\begin{array}{c} N-M\\ n-i \end{array}\right)}{\left(\begin{array}{c} N\\ n \end{array}\right)}$$

Here, in the equation, N is the number of all genes with GO annotations; n is the number of DES target genes in N; M is the number of all genes annotated with a specific GO term; and m is the number of DES target genes in M. The *P*-value was corrected by the Bonferroni method (Ludbrook, 1998); a corrected *P*-value ≤ 0.05 was taken as the threshold. GO terms fulfilling this condition were defined as significantly enriched.

#### Pathway Enrichment Analysis

KEGG (Kanehisa et al., 2007) was used to perform pathway enrichment analysis to identify significantly enriched metabolic or signal transduction pathways in DES target genes when compared with the whole genome.

The formula was as for GO analysis. N is the number of all genes with KEGG annotations; n is the number of DES target genes in N; M is the number of all genes annotated with a specific pathway; m is the number of DES target genes in M. The *P*-value was corrected by the Bonferroni method (Weisstein, 2004); a corrected *P*-value <0.05 was taken as the threshold. KEGG terms fulfilling this condition were defined as significantly enriched.

## Results

### General Data Information

The first results of our experiments turn out to be the evaluation of the guinea pig models used for further analyses. Based on the scoring criteria, we screened out qualified animals with a total score of ≥5 as candidate guinea pigs for further grouping and sequencing. Then, we sequenced the microRNAs from four group: model establishment group (negative control); models adding antagonists against HR4 (positive control); group P-ONE for models adding peptide P-ONE and group P-TWO for models adding peptide P-TWO. After sequencing and data preprocessing, we firstly summarized the numbers detected small non-coding RNAs from each group shown in Table 3. After such data, we can identify that:

**TABLE 3:** Summary of detected small non-coding RNAs for each sample.

Sample name	Known miRNA count	Novel miRNA count	Known piRNA count	Novel piRNA count	Known siRNA count	Novel siRNA count
Negative Control	266	1976	0	3467	0	0
Positive Control	264	1044	0	3618	0	0
P-ONE	317	768	0	1308	0	0
P-TWO	289	3294	0	26333	0	0

(1) Small non-coding RNAs (sncRNAs) have quite different distribution patterns in different samples, indicating their different biological status;
(2) Most of samples have similar number of known microRNAs, indicating effective microRNA may be stable and may not participate in related regulations;
(3) No siRNAs have been identified in all the samples.

**FIGURE 1:**
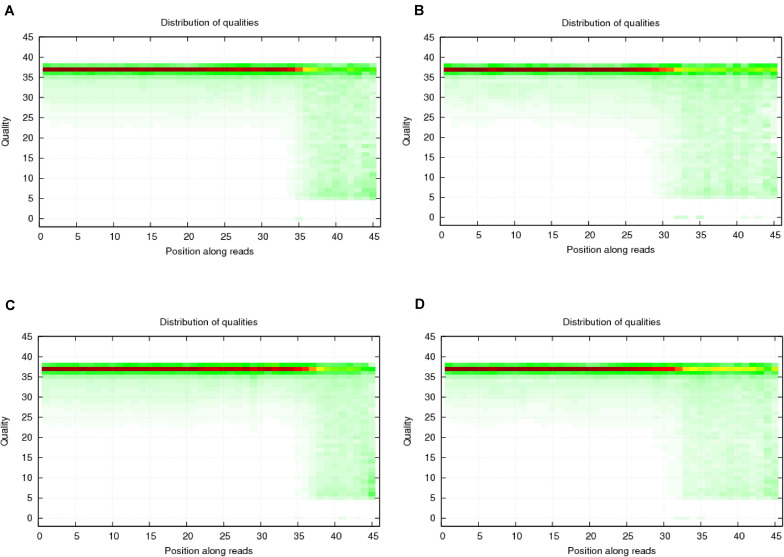
Quality distribution of four sequencing results. **(A)** Negative control group; **(B)** Positive control group; **(C)** P-ONE group; **(D)** P-TWO group. From such four bar plots, we can confirm that all sequencing results are of high quality (greater than 20), satisfying the requirements for further processing and analyses.

**FIGURE 2:**
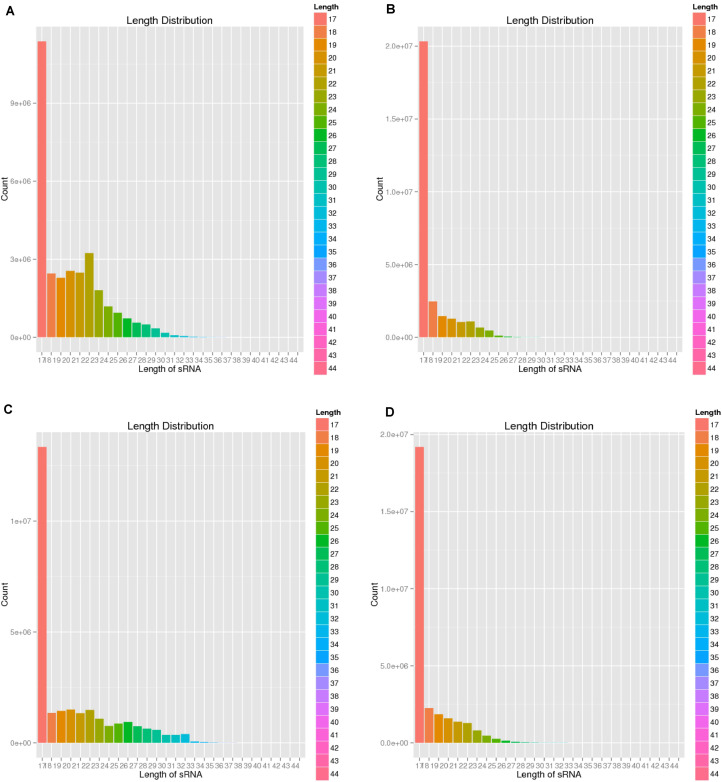
Length distribution of four sequencing results. **(A)** Negative control group; **(B)** Positive control group; **(C)** P-ONE group); **(D)** P-TWO group. From such four bar plots, most of the small RNAs have reasonable length less than 18 nt, corresponding with the general distribution of small RNAs’ length. Therefore, such results validated the high-quality of our sequencing and the accurate identification of small RNAs.

To verify the distribution pattern, the first step is to verify the quality of small RNA sequencing. Therefore, we firstly showed the sequencing qualities length distribution of small RNAs among different samples (Figures 1, 2). According to such two figures, it’s easy for us to confirm that:

(1) Our sequencing is of high quality among all the samples: generally, sequencing with unstable quality along the genomic position or with averaged quality lower than 20 are regarded as low quality sequencing data. Our sequencing data has a stable quality greater than 35, ensuring the reliability of our further analysis;
(2) The identification of small RNAs is quite effective using our experimental and computational methods;
(3) Such small RNA sequencing results can be processed for further analysis.

### Annotation of Small RNAs

**FIGURE 3:**
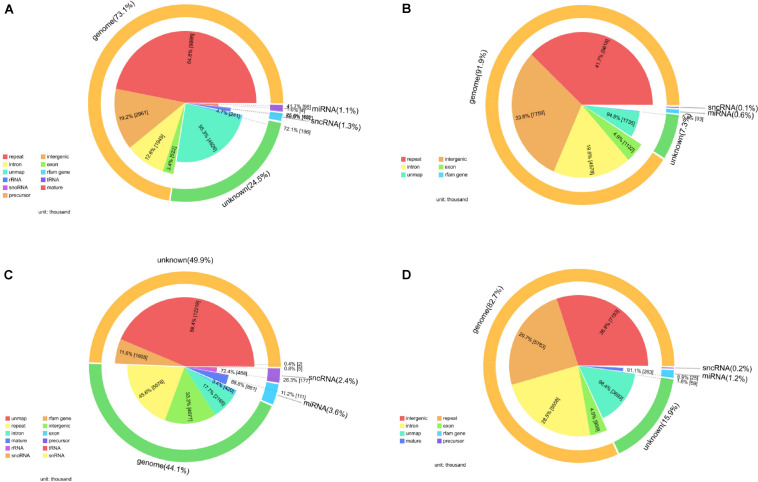
Catalog of small RNAs in four sequencing groups. **(A)** Negative control group; **(B)** positive control group; **(C)** P-ONE group; **(D)** P-TWO group. For all the four samples, most of the so-called small RNAs all come from the genome especially the intergenic regions and only about 5% of all the small RNAs can be defined as microRNA or small non-coding RNAs. And for all the samples, there still remain thousands of unknown small RNAs for further exploration.

After filtering, the next result obtained from analyses turned out to be the annotation name and genome locations of such identified small RNAs. Clean tags were mapped to sRNA database such as miRBase and Rfam. Table 4 lists separate mapping rate for each sample and Figure 3 shows the distribution of tags. The proportion of all kinds of sRNA is shown in Figure 3. According to Figure 3, different sample groups have quite different distribution of small RNA subtypes but they do share some specific prosperities:

**TABLE 4:** Summary of detected tags for each sample.

Sample name	Total tag	Mapped tag	Percentage (%)
Negative Control	21100237	15900803	75.36
Positive Control	25112110	23343614	92.96
P-ONE	27733405	14780247	53.29
P-TWO	23575452	19846022	84.18

(1) Most of the identified small RNAs can be mapped to the genome.
(2) There still remain various unknown small RNAs for further identification and function exploration with different proportions in different samples.
(3) Among those genomic derived small RNAs, most of such RNAs derived from genetic repeats and intergenic regions.

Based on the annotation of small RNAs, we summarized the number and distribution patterns of small RNAs that have already been confirmed and validated before, trying to reveal potential functional small RNA contribution on allergic rhinitis.

### Prediction of Unknown Small RNAs

After the annotation of small RNAs, there still remain a lot of unknown tags and small RNAs. Therefore, it’s quite necessary for us to identify new participators for the pathogenesis of allergic rhinitis at small RNA level. The identification/prediction of new small RNAs may not only help us enrich feature candidates for distribution comparison, but also predicted potential functional new small RNAs. Here, we used effective software : miRDeep2 (Friedländer et al., 2008) (for animals) and RIPmiR (Breakfield et al., 2012) (for plants) to predict some unknown small RNAs (microRNAs and piRNAs) based their architectural features.

### Expression Identification of Small RNAs

The small RNA expression level is calculated by using TPM, which is standardized for comparison.

### Target Prediction of MicroRNAs Using Two Typical Computational Software

**FIGURE 4:**
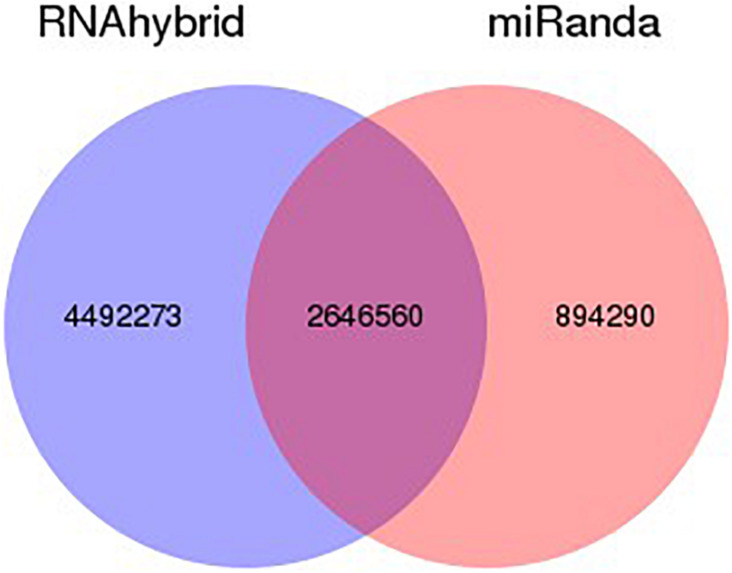
Venn statistics of filtered predicted targets. Using RNAhybrid and miRanda, we identified potential targets for our identified miRNAs. According to the prediction results, among all the predicted targets, most of the targets are shared by both RNAhybrid and miRanda. Comparing such two methods, RNAhybrid may work better with more unique prediction results.

The target gene/transcripts of microRNAs may actually reflect the biological functions and significance of microRNAs. We can use two effective software (RNAhybrid and miRanda) to get the target gene of miRNA, extract intersection or union of target gene as final prediction result. The combined target result as shows in Figure 4. According to the prediction results, RNAhybrid and miRanda shared various predicted targets (2646560), while RNAhybrid can identify more unique targets comparing to miRanda (4492273 vs 894290). The detailed distribution and comparison of such prediction results can be seen in Figure 4.

### Screening Differentially Expressed piRNAs

**FIGURE 5:**
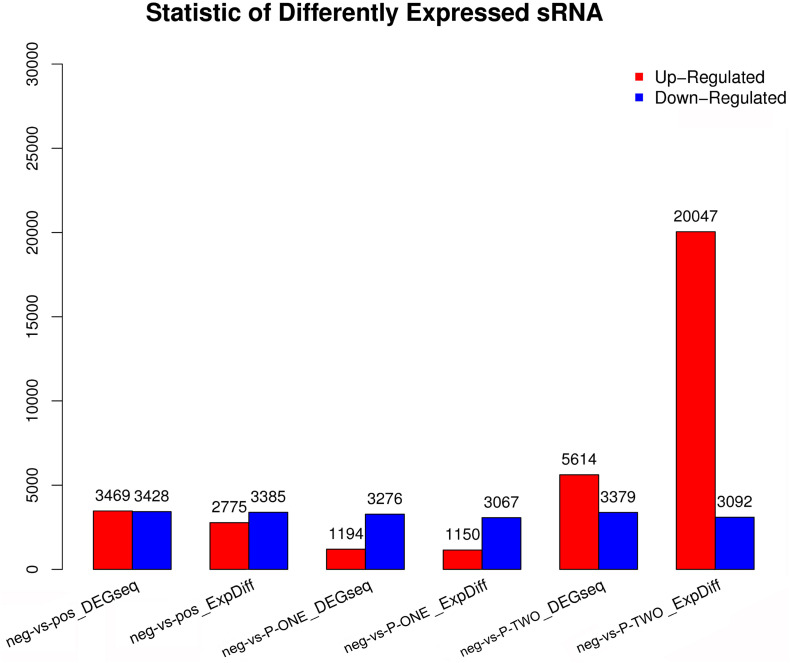
Statistic of differential expressed sRNAs. Using DEGseq nad ExpDiff, we identified the differentially expressed piRNAs from four groups: 1) negative control group (neg); 2) positive control group (pos); 3) P-ONE group; 4) P-TWO group. *X*-axis represents pairwise and *Y*-axis means number of screened DESs. Blue bar denotes down-regulated and orange bar for the up-regulated. According to the figure, comparing to the control group, P-TWO have quite a large group of upregulated pi-RNAs, indicating that P-TWO may have specific pharmacological effects via microRNA regulation.

Differentially Expressed small RNAs (DESs) screening is aimed to find differentially expressed small RNA between samples and do the further analysis. We use DEGseq and ExpDiff methods to do this analysis on piRNAs. The DESs in each pairwise as shown in Figure 5.

### Screening Differentially Expressed miRNAs

**FIGURE 6:**
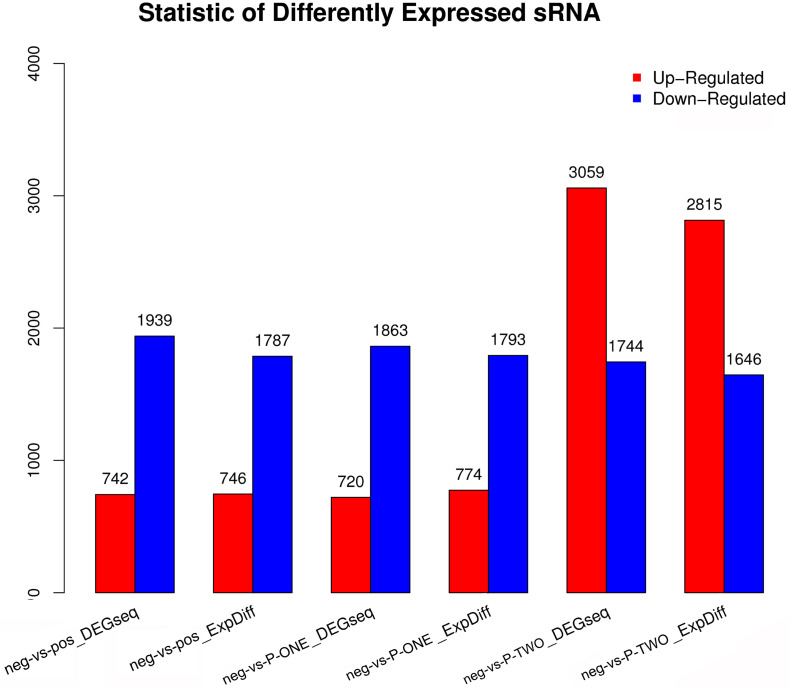
Statistic of differential expressed miRNAs. Using DEGseq nad ExpDiff, we identified the differentially expressed miRNAs from four gourps: 1) negative control group (neg); 2) positive control group (pos); 3) P-ONE group; 4) P-TWO group. *X*-axis represents pairwise and *Y*-axis means number of screened DESs. Blue bar denotes down-regulated and orange bar for the upregulated. According to the figure, P-ONE and traditional HR4-targeted method may have similar regulatory mechanisms via microRNAs but P-TWO may have its specific pharmacological effects and mechanisms via microRNA regulation.

Similar with the identification of differentially expressed miRNAs, using software like DEGseq and ExpDiff, we also identified differentially expressed microRNAs in different groups. The distribution of differentially expressed microRNAs in each pairwise as shown in Figure 6. According to the figure, we still focused on the differentially expressed miRNA pattern of With DESs, we perform hierarchical clustering of three comparisons: negative controls and P-ONE; negative controls and P-TWO and negative controls and positive controls. Based on such comparation, we identified various expression statistics at miRNA level:

(1) MicroRNAs have similar alteration pattern in positive controls and P-ONE group, implying that via microRNAs, the therapeutic mechanisms of P-ONE may share some specific regulatory processes with the traditional HR4-based therapeutics.
(2) However, P-TWO may have totally different regulatory mechanism considering its specific different alteration pattern comparing to P-ONE and positive control.

### DESs Target Prediction

As we have described in the Methods, we also identified some target of the DESs. The DESs target were performed by using several software.

### Gene Ontology Enrichment Analysis of DESs Targets

According to previous analyses, we identified thousands of genes targeted by differential expressed miRNAs. However, it’s impossible and unreasonable to analyze the biological effects of such genes one by one. To show the detailed correlations between genes targeted by differentially expressed microRNAs and AR therapeutic effects, here, we introduced gene ontology (The Gene Ontology Consortium, 2016) and KEGG terms (Kanehisa et al., 2016, 2018) to describe the functional distribution of such targeted genes.

**FIGURE 7:**
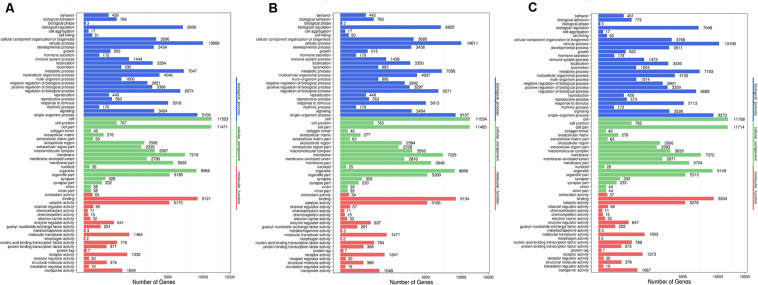
Go functional classification of DES target genes from three comparison. **(A)** Comparison between negative control and positive control. **(B)** Comparison between negative control and P-ONE. **(C)** Comparison between negative control and P-TWO. *X*-axis means number of DEGs (the number is presented by its square root value). *Y*-axis represents GO terms. All GO terms are grouped in to three ontologies: blue is for biological processes; brown is for cellular component and orange is for molecular functions. According to three plots, DES target genes may enrich in similar pattern under three therapeutic conditions, implying that such three therapeutic methods (HR4 antagonist, P-ONE and P-TWO) may have similar pharmacological mechanisms and microRNA may play an irreplaceable role during such processes.

Based on the methods we described in Methods, we further performed Gene Ontology (GO) enrichment analysis (The Gene Ontology Consortium, 2016) with screened DESs target genes. GO functional classification is listed to help understanding the distribution of gene functions of the specie from the macro level. To reveal the detailed pharmacological effects of P-ONE and P-TWO, we chose three comparison to show with GO functional classification box plot. The comparison can be seen in Figure 7. Comparing the GO classification box plot, it’s easy to find out that DES target genes may enrich in similar pattern under three therapeutic conditions, implying that such three therapeutic methods (HR4 antagonist, P-ONE and P-TWO) may have similar pharmacological mechanisms and microRNA may play an irreplaceable role during such processes.

### Pathway Enrichment Analysis of DESs Targets

**FIGURE 8:**
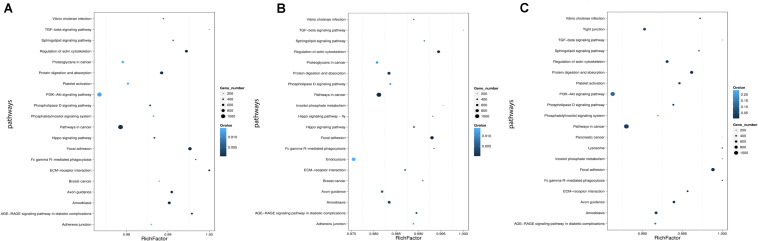
Statistics of pathway enrichment in each pairwise. **(A)** Comparison between negative control group and positive control group. **(B)** Comparison between negative control group and P-ONE. **(C)** Comparison between negative control group and P-TWO. Rich Factor is the ratio of DESs target genes numbers annotated in this pathway term to all gene numbers annotated in this pathway term. Greater Rich Fator means greater intensiveness. *Q*-value is corrected *P*-value ranging from 0 to 1, and less *Q*-value means greater intensiveness. We just display the top 20 of enriched pathway terms.

**FIGURE 9:**
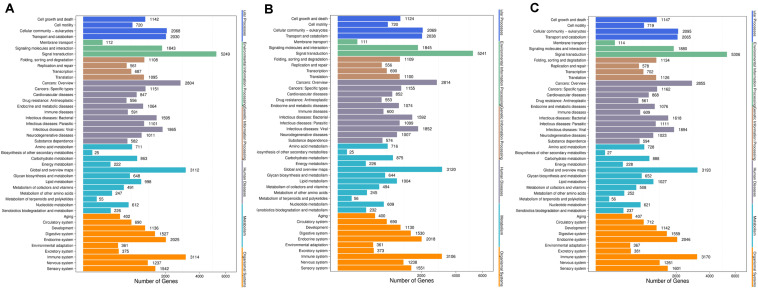
KEGG classification of each pairwise. **(A)** Comparison between negative control group and positive control group. **(B)** Comparison between negative control group and P-ONE. **(C)** Comparison between negative control group and P-TWO. *X*-axis means number of DEGs. *Y*-axis represents second KEGG pathway terms. All second pathway terms are grouped in top pathway terms indicated in different color.

Genes usually interact with each other to play roles in certain biological functions. We perform pathway enrichment analysis of DESs target genes based on KEGG database (Kanehisa et al., 2016, 2018) and generate a report for DESs target genes in each pairwise, respectively. In addition, we generate a scatter plot for the top 20 of KEGG enrichment results as Figure 8 and a bar plot for the statistics of KEGG terms types as Figure 9.

According to the KEGG enrichment figures, we can summarize the different functional enrichment pattern under three therapeutic conditions (HR4 antagonist, P-ONE and P-TWO):

(1) The detailed KEGG enrichment pattern under three conditions are different involving different regulatory pathways.
(2) Some specific pathways like pathways in cancer, TGF-beta signaling pathway and focal adhesion are shared in all the three groups, indicating the potential contribution of such pathways for the pharmacological effects of such three treatment methods.
(3) Still, there are various specific pathways that is differentially enriched in three groups. For instance, PI3K signaling pathway is only enriched in positive control group (HR4 antagonist) and P-TWO treatment group, but not P-ONE treatment group, revealing the potential differences among such three therapeutic methods.

## Discussion

Here, as we have presented above, we accomplished a systematic analysis on the small RNA (piRNAs and small RNAs) distribution pattern and potential targeting functional distribution pattern under different therapeutic conditions against AR. To further discuss the underlying therapeutic mechanisms of two reported epitopes against H4 receptor for allergic rhinitis treatment at the microRNA level and try to reveal their potential pharmacological mechanisms by targeting H4 receptors, we divided our discussion in two parts : (1) discussion on the differential small RNA distribution patterns; (2) discussion on functional clustering of genes targeted by the differential expressed microRNAs.

### Discussion on the Differential Small RNA Distribution Patterns

As we have shown in Figures 5, 6, it is obvious to see that at piRNA level, although there is differential expression patterns in P-ONE and P-TWO, however, the positive control does not show alterations at piRNA level, indicating that such alteration induced by P-ONE and P-TWO may not be directly correlated with targeting HR4 and therapeutic effects on AR. According to recent publications, no direct reports indicate that piRNAs may play effective role in the regulation of HR4 during the pathogenesis of AR, further explaining the specific pattern of piRNAs in the positive control groups. However, there are various publications, in deed confirmed that piRNAs may contribute to the pathogenesis of AR via some specific regulatory mechanisms like interacting with PTEN (Phosphatase and Tensin homolog) (Alexandrova et al., 2016) and PI3k (Phosphoinositide 3-kinase) (Alexandrova et al., 2016; Narożna et al., 2017). Considering that traditional HR4 antagonists only block HR4 by physical binding, however, our newly identified peptides block HR4 biological functions by triggering specific immune response against HR4. Therefore, although also targeting HR4, such two peptides may also have some unique therapeutic contributions on AR, probably via PTEN or PI3K associated biological processes. The detailed biological mechanisms may still need further molecular and cell biology studies to reveal. What’s more, actually, the distribution patterns of P-ONE and P-TWO are also quite different, P-TWO has greater effects on the regulation of piRNAs, indicating that such two peptides may still trigger different immune response and have different pharmacological mechanisms against AR.

Different from the distribution pattern of piRNAs, the distributions of miRNAs are quite similar between positive group and P-ONEp group, indicating that at miRNA level, such two methods may have similar therapeutic effects on AR. However, as for the P-TWO group, the distribution of up-regulated and down regulated microRNAs are reversed. More microRNAs turn out to be up-regulated in such pattern. Such phenotype cannot be explained now. However, at least, such results indicate that P-TWO has quite different pharmacological effects on microRNA level comparing to P-ONE and traditional HR4 antagonists.

All in all, summarized from such figures, we can conclude that:

(1) At piRNA level, P-ONE, P-TWO and traditional HR4 antagonists have totally different expression pattern, indicating their different regulatory effects and pharmacological mechanisms.
(2) At miRNA level, P-ONE may have similar therapeutic effects with HR4 antagonists but P-TWO has quite unique therapeutic effects on such level.
(3) The typical alteration of small RNA expression level confirmed that small RNAs in deed play an irreplaceable role during the therapy of AR and participate in the pharmacological mechanisms of such medicine.
(4) Also, in terms of methodology, software DEGseq and ExpDiff may have quite comparable results.

### Discussion on Functional Clustering of Genes Targeted by the Differential Expressed MicroRNAs

Apart from such phenotypic discussion on the expression comparison of small RNAs in different groups, using gene ontology and KEGG annotation and clustering, we also identified some specific enrichment patterns under different therapeutic conditions, helping reveal the potential pharmacological effects of P-ONE and P-TWO comparing to traditional HR4 antagonists.

Here, firstly, we focused on Figure 7 describing the results of gene ontology enrichment analyses. Based on the gene ontology classification, we can summarize that the microRNA target expression pattern is quite similar under such three treatment conditions. Therefore, according to such results, although some regulatory details of P-ONE and P-TWO are different from traditional HR4 antagonists, actually, the comprehensive regulatory effects of such two peptides may still be the same at microRNA regulatory level. Further, such results also confirmed that new drugs like P-ONE and P-TWO only affect similar biological processes comparing with previous HR4 antagonists. Therefore, such two peptides may also be safe to be used in further therapies.

Apart from gene ontology, we also focused on the KEGG annotation and clustering results. Based on Figure 8, we presented the pathway enrichment pattern in each pairwise. Here, we identified some specific KEGG pathways that differentially enriched in different experimental groups.

Firstly, there are still some shared KEGG pathways that have been identified under all the same conditions, indicating its specific role for AR pathogenesis and therapies at microRNA regulatory level. For instance, TGF-beta signaling pathway, according to recent publications, such biological process has been widely reported to be a specific pathological pathway for AR. Early in 1992, researchers in the United States have confirmed that TGF beta 1 as a core regulator in such signaling pathway contribute to the pathogenesis of chronically inflammation in human upper airway tissues, related to the onset of allergic rhinitis (Ohno et al., 1992). Further in 2002, another independent study further confirmed the pathogenesis of allergic rhinitis is directly correlated with TGF-beta effects (Benson et al., 2002). Therefore, the identification of such pathway by all the three therapeutic treatment confirmed that such two new medicine also relied on interfering one of the most significant pathways of AR to cure such disease. What’s more, more recent publications (Akdis et al., 2005; Jutel et al., 2006; Kucuksezer et al., 2013) on TGF-beta and allergic rhinitis also indicate that TGF-beta is associated with the abnormal immune responses of AR, corresponding with the designed principal of P-ONE and P-TWO which is triggering antigen-specific immune response against HR4.

Apart from such shared biological processes, we also identified some effective biological processes that is only recognized by P-ONE and P-TWO. For P-ONE, endocytosis is a unique pathway with quite low *Q*-value and has not been identified by group positive control and P-TWO. In 2019, a specific publication (Blanco-Pérez et al., 2019)confirmed that a unique pattern of endocytosis mediated allergen fusion contributing to the relief of specific allergies, implying that endocytosis may also contribute to the pathogenesis of AR. The functional enrichment of P-ONE associated microRNA targets may indicate that P-ONE may potential inhibit abnormal allergic effects by interfering allergen fusion, presenting a new theory for the pharmacological effects of P-ONE. Similarly, as for P-TWO, there are still some detailed biological processes and pathways that are uniquely identified in such group. For instance, the lysosome, although with a relatively high *q*-value, recent publications (Ring and Munehen, 1983; Kohno et al., 1987; Liu et al., 2005) also reported that such biological process also regulated the abnormal immune response of AR. In 2005, a specific histopathological study (Liu et al., 2005) on allergic rhinitis confirmed that another drug named as Centipeda minima treats AR by interfering lysosome associated biological processes. Therefore, the enrichment of microRNA targets in such biological process may indicate that P-TWO, our new peptide drug may interact with lysosome associated biological processes and interfere the pathogenesis of AR under certain mechanisms.

Further, we identified the KEGG classification pattern for each pairwise. Although we have identified various unique KEGG pathways for each comparison, the general classification pattern of such three pariwises are quite similar with each other, implying the general therapeutic effects contributed by microRNA regulation and the safety of our new drugs P-ONE and P-TWO.

All in all, as we have mentioned analyzed above, at the functional level, we can summarize that:

(1) Both P-ONE and P-TWO has similar general and comprehensive therapeutic effects comparing to traditional HR4 antagonists at microRNA regulation level according to gene ontology analyses.
(2) The general pharmacological effects of P-ONE and P-TWO are similar with those of traditional HR4 antagonists at microRNA regulatory level. Therefore, P-ONE and P-TWO may be safe to be applied in clinics considering its systematic effects in vivo.
(3) According to KEGG pathway enrichment analyses, there are still some differential regulatory effects of different treatment strategies at microRNA regulatory level. The biological foundations of differential therapeutic effects induced by P-ONE and P-TWO have all been supported by recent publications.
(4) Some specific pathways like endocytosis, lysosomes, hippo signaling pathway and inositol phosphate metabolism may be significant and specific pharmacological mechanisms for our new drugs P-ONE and P-TWO comparing with previously widely reported HR4 antagonists.

## Conclusion

Relied on stable AR models, we identified the pharmacological effects of our two new candidate peptide drugs P-ONE and P-TWO on the small RNA level comparing to traditional HR4 targeting antagonists. Based on the small RNA profiling results, we firstly confirmed that P-ONE, P-TWO and traditional HR4 targeting antagonists have specific therapeutic on AR at microRNA level. Apart from that, the comprehensive effects of such three treatment strategies are quite similar. For details, based on KEGG pathway enrichment analysis, we also identified some unique pharmacological effects of new drugs P-ONE and P-TWO. All in all, using small RNA sequencing techniques, for the first time, we compared the pharmacological effects of P-ONE, P-TWO and traditional drugs and revealed both the similarities and the differences of such strategies at small RNA regulatory level, laying a solid foundation for the comprehensive understanding of the new drugs’ pharmacological mechanisms and the potential pathogenesis of AR.

## Data Availability Statement

The datasets generated for this study can be found in NCBI SRA. The study number of SRA database is SRP278422 (https://trace.ncbi.nlm.nih.gov/Traces/sra/?study~=~SRP278422), and the BioProject number is PRJNA658395 (https://www.ncbi.nlm.nih.gov/bioproject/PRJNA658395).

## Ethics Statement

The animal study was reviewed and approved by the Institutional Animal Care and Use Committee (IACUC) of Jilin University approved all animal procedures [permit number: SYXK(2014-0012)].

## Author Contributions

LL: conception or design of the work. L-FA: data collection. Z-DL: data analysis and interpretation. L-FA, Z-DL, and LL: manuscript drafting and final approval of the version to be published. All authors contributed to the article and approved the submitted version.

## Conflict of Interest

The authors declare that the research was conducted in the absence of any commercial or financial relationships that could be construed as a potential conflict of interest.

## Footnotes

1 http://www.geneontology.org/

2 http://www.yeastgenome.org/help/analyze/go-term-finder
